# Intimal and medial calcification in relation to cardiovascular risk factors

**DOI:** 10.1371/journal.pone.0235228

**Published:** 2020-07-13

**Authors:** Sabine R. Zwakenberg, Pim A. de Jong, Eva J. Hendriks, Jan Westerink, Wilko Spiering, Gert J. de Borst, Maarten J. Cramer, Jonas W. Bartstra, Teddo Doesburg, Femke Rutters, Amber A. van der Heijden, Casper Schalkwijk, Leon J. Schurgers, Yvonne T. van der Schouw, Joline W. J. Beulens, on behalf of the SMART Study Group

**Affiliations:** 1 Julius Center for Health Sciences and Primary Care, University Medical Center Utrecht, Utrecht University, Utrecht, The Netherlands; 2 Department of Radiology, University Medical Center Utrecht, Utrecht University, Utrecht, The Netherlands; 3 Department of Vascular Medicine, University Medical Center Utrecht, Utrecht University, Utrecht, The Netherlands; 4 Department of Vascular Surgery, University Medical Center Utrecht, Utrecht University, Utrecht, The Netherlands; 5 Department of Cardiology, University Medical Center Utrecht, Utrecht University, Utrecht, The Netherlands; 6 Department of Radiology, Westfries Gasthuis, Hoorn, The Netherlands; 7 Department of Epidemiology & Biostatistics, Amsterdam Public Health research institute, Amsterdam UMC – Location VUmc, Amsterdam, The Netherlands; 8 Department of General Practice and Elderly Care Medicine, Amsterdam Public Health research institute, Amsterdam UMC – Location VUmc, Amsterdam, The Netherlands; 9 Department of Internal Medicine, Maastricht University Medical Centre, Maastricht, The Netherlands; 10 CARIM School for Cardiovascular Diseases, Maastricht University Medical Centre, Maastricht, The Netherlands; 11 Department of Biochemistry, Cardiovascular Research Institute Maastricht (CARIM), Maastricht University, Maastrich, The Netherlands; Université de Picardie Jules Verne, FRANCE

## Abstract

**Purpose:**

To assess specific risk factors and biomarkers associated with intimal arterial calcification (IAC) and medial arterial calcification (MAC).

**Methods:**

We conducted a cross-sectional study in patients with or at risk of vascular disease from the SMART study(n = 520) and the DCS cohort(n = 198). Non-contrast computed tomography scanning of the lower extremities was performed and calcification in the femoral and crural arteries was scored as absent, predominant IAC, predominant MAC or indistinguishable. Multinomial regression models were used to assess the associations between cardiovascular risk factors and calcification patterns. Biomarkers for inflammation, calcification and vitamin K status were measured in a subset of patients with IAC(n = 151) and MAC(n = 151).

**Results:**

Femoral calcification was found in 77% of the participants, of whom 38% had IAC, 28% had MAC and 11% were scored as indistinguishable. The absolute agreement between the femoral and crural arteries was high(69%). Higher age, male sex, statin use and history of coronary artery disease were associated with higher prevalences of femoral IAC and MAC compared to absence of calcification. Smoking and low ankle-brachial-index (ABI) were associated with higher prevalence of IAC and high ABI was associated with less IAC. Compared to patients with IAC, patients with MAC more often had diabetes, have a high ABI and were less often smokers. Inactive Matrix-Gla Protein was associated with increased MAC prevalence, while osteonectin was associated with decreased risk of MAC, compared to IAC.

**Conclusions:**

When femoral calcification is present, the majority of the patients have IAC or MAC throughout the lower extremity, which have different associated risk factor profiles.

## Background

Arterial calcification is associated with increased cardiovascular risks [1]. Intimal arterial calcification (IAC), considered a representation of underlying atherosclerotic plaque burden, has long been held responsible for this association, whereas other types of arterial calcification were often considered innocent [2]. In recent years, increasing interest is directed towards medial arterial calcification (MAC) and other forms of non-atherosclerotic calcification, such as calcification of the internal elastic lamina [2].

Based on radiology-pathology correlation studies, IAC is thought to have a spotty/patchy picture on radiographs, whereas MAC and internal elastic lamina calcification tend to outline the artery with a smooth layer of calcium, resulting in linear or annular calcifications on radiographs [3,4]. These calcification patterns may represent different pathophysiological mechanisms driven by separate risk factors, subsequently leading to different forms of cardiovascular disease (CVD). While IAC is thought to be characterized by inflammation and lipid deposition in plaques, MAC is suggested to reflect a process of active calcification resembling bone formation [5–8].

Several studies have related MAC to risk factors and outcomes [6–11]. These studies found that advanced age [7–10], diabetes [6,8–10], and kidney disease [6,12] are risk factors predominantly associated with MAC, and despite some heterogeneity, most studies report no associations between MAC and hypertension or dyslipidaemia [6–9]. Studies on IAC, primarily related to coronary arteries, report positive associations with traditional cardiovascular risk factors, including smoking and dyslipidaemia [6,8,13]. However, the available information is limited as most of these studies were performed in selected groups, such as patients with diabetes and chronic kidney disease, and only two studies assessed both MAC and IAC [6,8].

Firstly, we aim to assess the prevalence of predominant IAC and predominant MAC, measured in the lower extremity arteries using computed tomography (CT), and the association with cardiovascular risk factor profiles in patients with or at high risk of vascular disease. Secondly, we aim to identify whether markers for calcification, inflammation and vitamin K status are potential biomarkers for MAC compared to IAC.

## Methods

### Study population

This study included participants of two ongoing cohort studies, the Second Manifestations of ARTerial disease (SMART) study [13] and the Hoorn Diabetes Care System (DCS) cohort [14]. This sub-study was approved by the medical ethics review board at the University Medical Center Utrecht (14/444), and all participants gave their written informed consent. Only patients with bilateral lower limb amputations were excluded.

The SMART study is an on-going prospective cohort study comprising patients with cardiovascular disease (CVD) and patients at high risk for CVD due to hypertension, diabetes or other cardiovascular risk factors. Details of the study have been described elsewhere [13]. Patients that were newly referred to the University Medical Center Utrecht with either manifest vascular disease or important cardiovascular risk factors were asked to participate. Newly referred patients, from March 2015 till December 2017, were invited for an additional unenhanced thin-slice CT scan of the legs, of which 548 participants agreed to participate and 520 participants signed the informed consent form and underwent the CT scan.

Participants with type 2 diabetes from all 103 general practitioners in the West-Friesland region in the Netherlands have been included in the Hoorn Diabetes Care System (DCS) cohort [14]. All participants are monitored annually and the clinical measures, obtained during the standardized examinations, are used for clinical research. Participants were approached individually for specific research projects. Details of this study have been described previously [14]. Participants with an annual visit between June 2017 and February 2018 were invited for an additional CT scan of the legs. In total, 200 participants agreed to participate of whom 198 participants signed the informed consent and underwent the CT scan.

### Calcification

All participants underwent an unenhanced CT-scan, scanning the legs from the femoral head to the feet. Slice thickness was 1 mm with a 0.7 mm increment. A semi quantitative CT score based on annularity, thickness and continuity of the calcifications was used according to a previously developed algorithm [15]. The score combined circularity (absent, dot(s), <90°, 90–270° or 270–360°), thickness (absent, ≥1.5 mm or <1.5 mm), and morphology (indistinguishable, irregular/patchy or continuous), where high degree of circularity, thin and continuous calcifications had a high likelihood of being predominant medial calcification, while low degree of circularity, thick and patchy calcifications had a high likelihood of being predominant IAC. When there was no calcification noticeable, the calcification was scored as “Absent”. When morphology was judged as indistinguishable (mainly in the case of scarce dots of calcification), the calcification was scored as “Indistinguishable”. In all other cases, points assigned in the scoring system (see S1 Table) were summed. Less than 7 points was defined as “Dominant IAC” and 7 or more points was defined as “Dominant MAC” [15]. A histogram of this score is shown in S1 Fig. Femoral and crural arteries were scored separately. All CT-scans were scored by an expert in reading CT-scans (PdJ), blinded for patient’s characteristics.

The algorithm was developed and validated in the intracranial internal carotid artery. The inter-rater reliability and intra-rater reliability for the algorithm was 0.72 (0.60–0.84) and 0.82 (0.73–0.89), respectively [14]. As it is not feasible to perform such an autopsy study in a large sample, the sample size of 16 did not allow for quantitative derivation of the optimal cutoff for medial calcification. Therefore, the cutoff was based on the characteristics that contribute to the different forms of calcification. Recently, the calcification score was validated using histological samples of the crural arteries (n = 96) [16]. 70% of the arteries were correctly classified as no calcification, predominant IAC or predominant MAC, resulting in an absolute agreement of 0.47 for IAC and 0.42 for MAC [16].

### Risk factor measurement

In SMART, all patients underwent a comprehensive screening at baseline, including questionnaires on medical history and medication use. In DCS, this information was extracted from the medical records, which were obtained during the annual visits. Medical history was categorized as coronary artery disease (CAD), cerebrovascular disease (CVA), aneurysm abdominal aorta (AAA) and peripheral arterial disease (PAD), which was diagnosed as Fontaine stage II-IV. In both cohorts, smoking was self-reported and classified as never, former and current smoker. Pack years was assessed in the SMART cohort only.

Physical examinations were performed including weight, height and blood pressure measurement (in seated position). Fasting blood samples were available for measurement of blood lipids, HbA1c, C-reactive protein (CRP) and creatinine levels. Glomerular filtration rate (eGFR) was estimated using the CKD-EPI formula [17]. Hypertension was defined as a systolic blood pressure (SBP) of ≥140 mmHg, a diastolic blood pressure (DBP) ≥90 mmHg and/or use of antihypertensive medication. The SMART definition of hyperlipidemia was used and defined as total cholesterol ≥5 mmol/L, LDL-cholesterol ≥3.2 mmol/L and/or use of lipid-lowering medication.

SMART participants were diagnosed with diabetes mellitus when there was a referral diagnosis of diabetes mellitus, self-reported diabetes mellitus, the use of glucose-lowering agents and/or a baseline fasting plasma glucose ≥7 mmol/L and a definitive diagnosis of diabetes during the first year of follow-up. In the DCS cohort, type 2 diabetes mellitus was reported when a patient had at least one or more classic symptoms and fasting plasma glucose ≥7.0 mmol/L or random plasma glucose ≥11.1 mmol/L or in the absence of symptoms, two elevated fasting plasma glucose concentration on two different time points when no symptoms are present.

Ankle brachial index (ABI) measurements were conducted by experienced professionals, using a Vasoguard dopplerprobe (8MHz). For each ankle, the highest SBP of the posterior tibial and dorsalis pedis (both measured twice) was used for the ABI calculations. An average SBP was calculated for each arm from at least 2 measurements of the brachial artery. The arm with the highest average was used for the ABI calculations. The leg-specific ABIs were thus calculated by dividing the highest average arm SBP by the highest of the ankle pressures of that leg. Finally, ABI was classified as low (≤0.9) or high (≥1.3) [18].

### Biomarkers

Biochemical measurements were performed in subgroups of patients with either predominantly IAC (n = 151) or predominantly MAC (n = 151). We selected all subjects with IAC in both the femoral and crural arteries. Of the subjects with MAC, only 120 participants had predominant MAC in both the femoral and crural arteries. Therefore, we added participants with femoral MAC, but absent (n = 11) or indistinguishable calcification (n = 20) in the crural arteries to the MAC selection. Inflammatory biomarkers, IL-6, IL-8 and TNF-α, were measured in serum with commercially available 4-plex sandwich immunoassay kits (Meso Scale Discovery (MSD), Rockville, MD, US). Calcification markers, osteocalcin, osteonectin and osteopontin, were measured in serum using a multiplex Human Bone Marker Panel II Kit of Meso Scale Discovery (MSD, Rockville MD, USA). The inter-assay and intra-assay variation coefficients were reasonable: 13.4% and 14.6% for IL-6, 14.1% and 8.6% for IL-8 and 9.8% and 9.0% for TNF-α. For osteocalcin, osteonectin and osteopontin the inter- and intra assay variants were good at 2.7%, 7.1%, 8.3%, 13.6%, 5.7% and 2.9% respectively.

Additionally, markers for vitamin K status were measured. Circulating dp-ucMGP levels were determined in EDTA plasma, using the commercially available IVD CE marked chemiluminescent InaKif MGP assay on the IDS-iSYS system (IDS, Boldon, UK). The within-run and total precision of this assay were 0.8–6.2% and 3.0–8.2%, respectively. The assay measuring range is between 300–12,000 pmol/L and was found to be linear up to 11,651 pmol/L. Circulating PIVKA-II (ucFII), or protein induced by vitamin K absence/antagonism II, levels were measured using a conformation-specific monoclonal antibody in an ELISA-based assay [19]. Results were expressed as arbitrary units per liter (AU/l), because in states of vitamin K deficiency circulating ucFII may comprise multiple forms of partially carboxylated FII and neither their relative abundance in serum nor their relative affinity for the antibody is known. Using electrophoretic techniques 1 AU is equivalent to 1 μg of purified ucFII [20]. The detection limit was 0.25 AU/ml ucFII in serum.

### Statistical analyses

Baseline characteristics were presented as means with standard deviations (SD) or medians with interquartile ranges (IQR) (in case of non-normally distributed variables) for continuous variables or proportions with number of cases for categorical variables, for SMART and DCS separately. Baseline variables were complete for 95% of the study population. Additionally, baseline characteristics were calculated according to the categories of femoral and crural calcification. Absolute level of agreement between the femoral and crural scores were calculated using a formula described by De Vet et al [21]. This formula was used since we wanted to estimate the absolute level of agreement rather than the relative measure, like the Cohen’s Kappa.

Risk factors for the different types of calcification were assessed, using multinomial logistic regression analyses, adjusted for age and sex and analyzed in strata of cohort. Presence of diabetes and pack years was assessed in the SMART cohort only. We assessed the risk of predominant IAC, predominant MAC and indistinguishable calcification with no calcification as reference category. To assess whether risk factors for predominant MAC differed from predominant IAC, we additionally performed logistic regression analyses, using MAC as outcome measurement with IAC as reference category, adjusted for age and sex and analyzed in strata of cohort. In sensitivity analyses, we investigated whether our results were robust to the proposed cutoff of 7 for determination of medial calcification by analyzing the data using a cutoff of 6 and 8 for medial calcification in the femoral artery.

Furthermore, we calculated means with standard deviations or medians and interquartile ranges for the biomarkers for the group with predominant IAC and MAC. Logistic regression analyses for the association between the biomarkers and MAC, compared to IAC were performed adjusted for age and sex and analyzed in strata of cohort.

All statistical analyses were performed using R, version 3.1.0 (R Foundation for Statistical Computing, Vienna, Austria.). Add-on package ‘nnet’ was used for multinomial regression analyses. A p-value of lower then 0.05 was considered statistically significant.

## Results

The SMART cohort consisted of 81% males, with a mean age of 59.7 ± 10.3 years, while 66% was male in the DCS cohort with a mean age of 67.9 ± 9.1 years (Table 1). Baseline characteristics according to the calcification categorization are presented in S2 Table (by femoral score) and S3 Table (by crural score).

**Table 1:** Baseline characteristics of the SMART (n = 520) and DCS cohort (n = 198).

	*SMART (n = 520)*	*DCS (n = 198)*
Age _(years)_	59.7 ± 10.3	67.9 ± 9.2
Male sex	422 (81%)	130 (66%)
BMI _(kg/m2)_	27.3 ± 3.9	30.1 ± 5.1
Diabetes (type 1 or 2)	81 (16%)	198 (100%)
Type 2 diabetes	78 (15%)	198 (100%)
Hypertension	264 (51%)	164 (83%)
Hyperlipidemia	70 (14%)	153 (77%)
Systolic blood pressure _(mmHg)_	131 ± 16	138 ± 19
Diastolic blood pressure _(mmHg)_	78 ± 10	79 ± 8
Smoking		
Current	113 (22%)	23 (12%)
Former	248 (48%)	115 (58%)
Never	158 (30%)	59 (30%)
High ABI _(>1.3)_	92 (18%)	51 (26%)
Low ABI _(<0.9)_	36 (7%)	10 (5%)
Statin use	430 (83%)	150 (76%)
*Manifest cardiovascular disease*		
Cerebrovascular disease	98 (19%)	9 (5%)
Coronary artery disease	384 (74%)	24 (12%)
Aneurysm abdominal aorta	24 (5%)	2 (1%)
Peripheral artery disease	31 (6%)	6 (3%)
eGFR _(ml/min/1.73m2)_	89 ± 23	75 ± 27
Triglycerides _(mmol/L)_	1.3 (1.0–1.8)	1.6 (1.1–2.1)
Total cholesterol _(mmol/L)_	4.4 ± 1.1	4.1 ± 1.0
LDL-cholesterol _(mmol/L)_	2.5 ± 0.9	2.1 ± 0.8
HDL-cholesterol _(mmol/L)_	1.2 ± 0.3	1.2 ± 0.4
HbA1c _(mmol/mol)_	38.3 ± 7.6	54.0 ± 12.3
CRP _(mg/L)_	1.6 (0.8–3.4)	1.8 (0.9–4.2)

Baseline characteristics are described as mean ± standard deviation, median (interquartile range) or number of participants (%). BMI: body mass index, bp: blood pressure, ABI: ankle brachial index, eGFR: estimated glomerular filtration rate, LDL: low-density lipoprotein, HDL: high-density lipoprotein, CRP: C—reactive protein.

**Fig 1:**
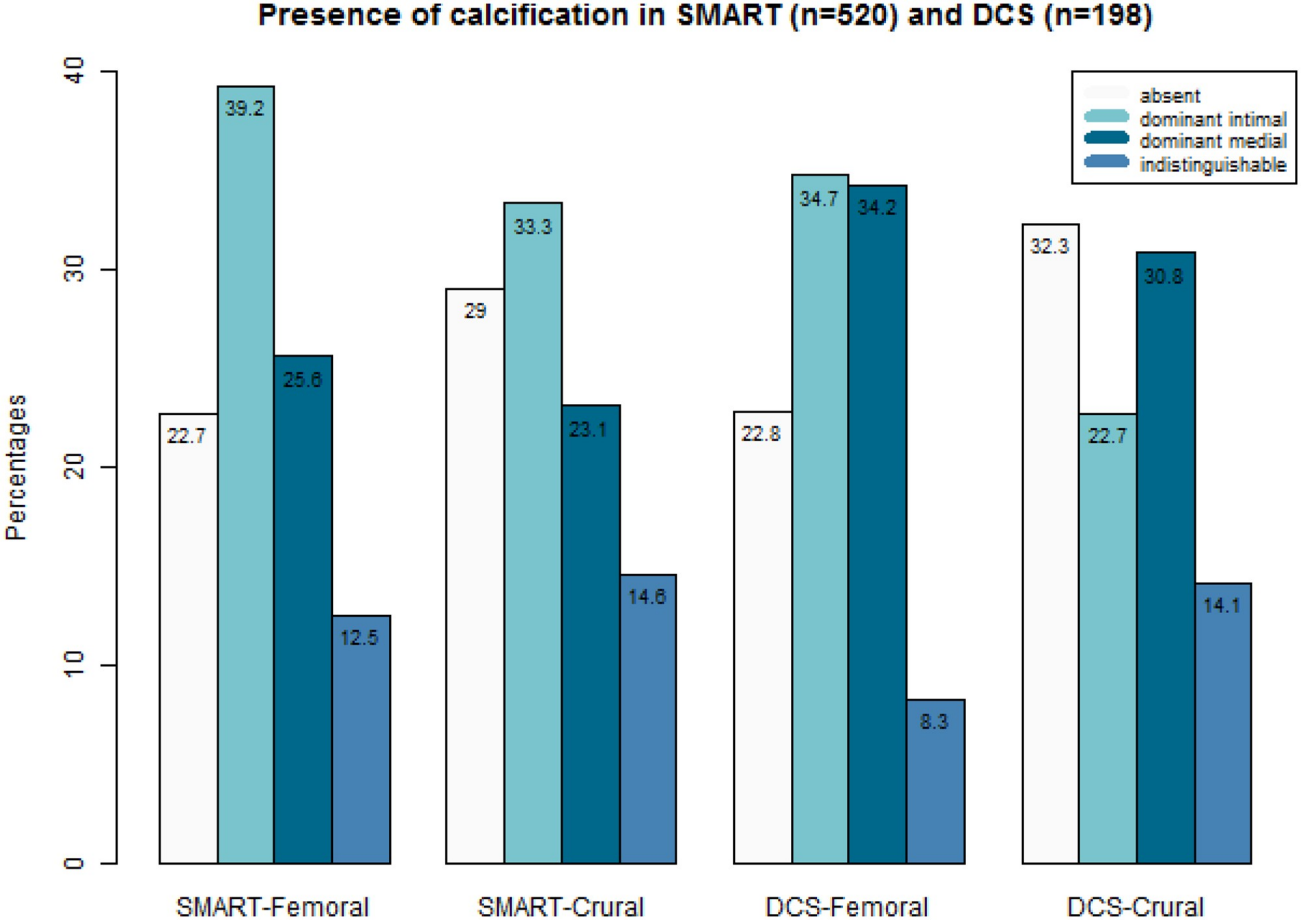
Frequencies of type of calcification in the femoral and crural arteries in the SMART (n = 520) and DCS (n = 198) cohort.

In the SMART cohort (n = 520), 39% (n = 204) had a predominant IAC pattern in the femoral arteries, while 26% (n = 133) of the participants were identified as predominant MAC (Fig 1). In the DCS cohort (n = 193 femoral, 198 crural), 35% (n = 67) had predominant IAC calcification, while 34% (n = 66) had predominant MAC in the femoral artery (Fig 1). In both SMART and DCS, 23% had no calcification (Fig 1). In the DCS cohort, the prevalence of predominant MAC in the crural arteries (31%) was similar to the femoral artery in the same cohort, but the prevalence of predominant IAC in the crural arteries (23%) was relatively low in this cohort. Thus, when femoral calcification was present, almost 85% of patients had a predominant arterial calcification type throughout the lower extremity (11% indistinguishable calcification /(100%-23% with no calcification) = 14,3%). We observed good absolute agreement across the crural and femoral arteries, as 69% of the patients with predominant IAC in the femoral arteries, also had predominant IAC in the crural arteries. The agreement was the same for MAC.

### Cardiovascular risk factors compared to absence of calcification

Risk factors for femoral calcification are presented in Table 2. Higher age, male sex, statin use and history of CAD were associated with both IAC and MAC compared to absence of calcification. Current smoking was associated with a higher prevalence of predominant IAC (OR 6.47 (3.28;12.79)), but no association was found for MAC. High ABI (≥1.3) was associated with less IAC (OR 0.33 (0.18;0.59), whereas low ABI (≤ 0.9) was associated with higher prevalence of predominant IAC (OR 6.76 (2.04;22.41). Higher HbA1c (OR 0.72 (0.58;0.90)) and history of CVA (OR 0.43 (0.24;0.79)) was associated with decreased prevalence of IAC.

**Table 2:** Risk (OR 95%CI) of predominant intimal, predominant medial or indistinguishable calcification compared to no calcification in the femoral artery.

	*Absent (n = 162)*	*Intimal (n = 271)*	*Medial (n = 199)*	*Indistinguishable (n = 81)*	*Media vs*. *Intimal*
Age _(per 10 years)_	1	3.02 (2.39;3.82)	3.37 (2.61;4.34)	1.68 (1.28;2.20)	1.03 (0.83;1.29)
Male sex	1	3.71 (2.25;6.12)	4.05 (2.34;7.00)	1.70 (0.94;3.06)	1.16 (0.70;1.93)
BMI _(perkg/m2)_	1	0.99 (0.94;1.04)	1.01 (0.96;1.06)	1.01 (0.95;1.07)	1.01 (0.97;1.06)
Diabetes _(type 1 and 2)_ ^#^	1	0.83 (0.40;1.71)	1.59 (0.76;3.33)	0.46 (0.17;1.26)	1.81 (1.03;3.15)
Hypertension _(yes vs no)_	1	0.88 (0.56;1.38)	1.18 (0.73;1.93)	0.90 (0.51;1.59)	1.24 (0.83;1.87)
Hyperlipidemia _(yes vs no)_	1	0.87 (0.54;1.42)	0.88 (0.52;1.47)	0.86 (0.46;1.59)	0.65 (0.38;1.13)
Systolic blood pressure _(per 10 mmHg)_	1	1.01 (0.89;1.16)	1.03 (0.89;1.19)	1.08 (0.91;1.28)	1.00 (0.90;1.12)
Diastolic blood pressure _(per 10 mmHg)_	1	0.91 (0.72;1.16)	0.86 (0.67;1.11)	1.17 (0.88;1.55)	0.94 (0.76;1.16)
Smoking _(current vs never)_	1	6.47 (3.28;12.79)	0.74 (0.35;1.58)	2.12 (0.96;4.71)	0.11 (0.05;0.21)
Pack years ^#^	1	1.04 (1.02;1.06)	1.00 (0.98;1.02)	1.03 (1.00;1.05)	0.96 (0.95;0.98)
High ABI _(>1.3)_	1	0.33 (0.18;0.59)	0.97 (0.56;1.69)	1.07 (0.57;2.04)	2.91 (1.78;4.76)
Low ABI _(<0.9)_	1	6.76 (2.04;22.38)	2.39 (0.63;9.05)	0.58 (0.06;5.45)	0.36 (0.17;0.78)
Statin use _(yes vs no)_	1	3.11 (1.79;5.41)	1.97 (1.12;3.46)	1.81 (0.94;3.49)	0.69 (0.39;1.15)
Manifest cardiovascular disease _(yes vs no)_					
Cerebrovascular disease	1	0.43 (0.24;0.79)	0.57 (0.31;1.07)	0.57 (0.28;1.18)	1.37 (0.76;2.48)
Coronary artery disease	1	3.31 (2.06;5.31)	2.54 (1.54;4.17)	2.06 (1.16;3.67)	0.93 (0.57;1.52)
Aneurysm abdominal aorta	1	3.05 (0.72;12.90)	1.49 (0.30;7.27)	0.68 (0.07;6.94)	0.52 (0.20;1.37)
Peripheral artery disease	1	2.42 (0.90;6.50)	0.80 (0.23;2.80)	1.28 (0.36;4.59)	0.34 (0.12;0.92)
eGFR _(ml/min/1.73m2)_	1	0.98 (0.89;1.09)	0.95 (0.85;1.05)	0.97 (0.86;1.10)	0.95 (0.88;1.03)
Triglycerides _(mmol/L)_	1	0.91 (0.76;1.08)	0.87 (0.72;1.06)	0.77 (0.58;1.03)	0.95 (0.80;1.13)
Total cholesterol _(mmol/L)_	1	1.01 (0.82;1.22)	0.87 (0.70;1.08)	1.01 (0.79;1.29)	0.88 (0.72;1.06)
LDL-cholesterol _(mmol/L)_	1	0.99 (0.78;1.26)	0.82 (0.63;1.08)	1.03 (0.77;1.38)	0.85 (0.67;1.08)
HDL-cholesterol _(mmol/L)_	1	2.22 (1.07;4.61)	2.07 (0.95;4.49)	2.75 (1.17;6.44)	0.97 (0.54;1.77)
HbA1c _(mmol/mol)_	1	0.72 (0.58;0.90)	0.98 (0.79;1.21)	0.74 (0.55;1.00)	1.37 (1.08;1.73)
CRP _(mg/L)_	1	1.00 (0.97;1.04)	0.99 (0.95;1.03)	0.96 (0.90;1.03)	0.99 (0.96;1.02)

Every line of this table represents a separate multinomial model. All models are adjusted for age and sex.

BMI: body mass index, bp: blood pressure, ABI: ankle brachial index, eGFR: estimated glomerular filtration rate,

LDL: low-density lipoprotein, HDL: high-density lipoprotein, CRP: c-reactive protein, AAA: Aneurysm abdominal aorta.

^#^ assessed in the SMART cohort only (intimal n = 208, medial n = 133, indistinguishable n = 66).

Comparable results were found for crural calcification (S4 Table). In contrast to the arteria femoralis, higher HDL-cholesterol levels and a history of CVA were not associated with IAC in the crural arteries. However, higher DBP seemed to decrease the prevalence of predominant MAC (OR 0.70 (0.54; 0.90) per 10 mmHg increase) in the crural arteries. In sensitivity analyses using cutoffs of 6 and 8 to determine medial calcification in the femoral artery, the same determinants were found (S5 and S6 Tables).

### Cardiovascular risk factors for medial calcification compared to intimal calcification

Compared to the participants with predominant IAC, diabetes (OR 1.81 (1.03;3.15)), HbA1c (OR 1.37 (1.08;1.73)) and an elevated ABI (OR 2.91 (1.78;4.76)) were associated with higher prevalence of predominant MAC in the femoral artery (Table 2). Smoking (both status and pack years), low ABI and history of PAD were associated with a lower prevalence of MAC.

In the crural arteries, male sex and BMI were associated with an increased prevalence of MAC compared to IAC (S4 Table). The association between low ABI and increased prevalence of MAC was no longer significant in the crural arteries, as well as the association between history of PAD and MAC. However, history of CAD and statin use were associated with a decreased prevalence of MAC compared to IAC in the crural arteries. In sensitivity analyses using cutoffs of 6 and 8 to determine medial calcification in the femoral artery, the same determinants were found (S5 and S6 Tables).

### Biomarkers for intimal and medial calcification

The median dp-ucMGP levels were slightly higher in participants with predominant MAC (686 pmol/L (IQR:495–853)), compared to IAC (466 pmol/L (IQR:469–742)) (S7 Table). Age and sex adjusted logistic regression analyses showed that increased dp-ucMGP tended to be associated with higher prevalence of MAC (OR 1.04 _per 100 pmol/L_ (0.99; 1.09) (Table 3). Increased osteonectin was associated with lower prevalence of MAC compared to IAC in the femoral artery (OR _per 100ng/ml_ 0.91 (0.83; 0.99)). Similar associations were observed in the crural arteries (Table 3).

**Table 3:** Results of multiple biomarkers and risk of medial (n = 151) compared to intimal calcification (n = 151) in the femoral and crural arteries.

	Femoral calcification	Crural calcification
*Vitamin K markers*	Continuous	
Dp-ucMGP _(pmol/L)_	1.04 (0.99;1.09) ^a^	1.05 (1.00;1.11)
PIVKA II >0.25 AU/ml*	1.25 (0.61;2.44)	1.25 (0.61;2.53)
*Bone markers*		
Osteocalcin _(ng/ml)_	1.00 (0.94;1.06) ^b^	0.99 (0.93;1.06)
Osteonectin _(ng/ml)_	0.91 (0.83;0.99) ^a^	0.91 (0.83;1.00)
Osteopontin _(ng/ml)_	1.02 (0.98;1.05)	1.01 (0.97;1.05)
*Inflammatory markers*		
IL-6 _(pg/ml)_	1.02 (0.98;1.05)	1.02 (0.98;1.06)
IL-8 _(pg/ml)_	1.00 (0.98;1.02)	0.99 (0.97;1.01)
TNF-alfa _(pg/ml)_	1.24 (0.94;1.64)	1.22 (0.92;1.60)

Models are adjusted for age and sex.

Dp-ucMGP: desphosphorylated-uncarboxylated Matrix Gla Protein, PIVKA-II: protein induced by vitamin K absence/antagonism II, IL: interleukin

^a^ per 100 increment,

^b^ per 5 increment,

* PIVKA is measured as detectable/non detectable with a detection limit of 0.25 AU/ml

## Discussion

When calcification is present, 85% of the patients have a predominant arterial calcification type (IAC or MAC) throughout the lower extremity based on our CT score. Cardiovascular risk factors appeared to differ between these calcification patterns. When comparing MAC to IAC, diabetes and high ABI were associated with a higher risk of MAC, while smoking and low ABI were associated with a higher risk of IAC. Furthermore, increased osteonectin levels were associated with a higher prevalence of IAC, while increased dp-ucMGP levels may result in higher prevalence of MAC.

Strengths of this study include the concurrent assessment of MAC and IAC and the comprehensive, standardized risk factor assessment performed in all patients included. Although we had to rely on a surrogate endpoint as histology was not feasible, the method to score intimal and medial calcification was validated histopathologically in the crural arteries, and showed a reasonable agreement [22]. However, a number of limitations apply to this study. The large proportion of patients that already have a cardiovascular diagnosis (SMART) and type 2 diabetes (DCS) in the study population is inherent to the sampling for this study. Selection conditional on having clinically manifest disease means that risk factors associated with this disease may show different interrelationships when studied amongst this selection compared to amongst the general population. Moreover, the results for the biomarkers are exploratory in nature because of the small sample size, and selection of biomarkers.

Vascular calcification is highly prevalent (77%) and patients have a predominant arterial calcification type throughout the lower extremity, 38% had predominant IAC, 28% had predominant MAC. Only two studies investigated the prevalence of IAC and showed a prevalence of 26.7% in patients with diabetes and 37.0% in hemodialysis patients [6,8]. In previous studies prevalence estimates of MAC in the lower extremities varied between 4.4–15.6% in healthy subjects [9,10], and 15.7%–41.5% for patients with diabetes [8–10], which is consistent with our findings. MAC is often ignored or considered innocent; however, this calcification type is highly prevalent, especially in diabetes patients.

People with type 2 diabetes could be considered as a target population for MAC, while smokers are at risk for IAC [6,8,10,16]. In patients with chronic kidney disease (CKD), renal function decline is a risk factor for MAC [6]. We could not confirm this association, which could be explained by the limited number of participants with a low renal function or renal function might not be a risk factor in patients with cardiovascular disease or patients at high risk of vascular disease. Consistent with our study, a study in people with type 2 diabetes (n = 1,059) showed that advanced age and duration of diabetes were risk factors for both IAC and MAC in the femoral arteries, whereas patients with IAC were more often smokers compared to patients without IAC [8]. Furthermore, HbA1c levels were higher in patients with MAC compared to patients without MAC [8]. LDL cholesterol is seen as a classic determinant of IAC in CKD patients [6]. However, the previous study including diabetes patients showed lower HDL cholesterol levels in patients with IAC [8], while we found higher HDL cholesterol levels in patients with femoral IAC. This might be explained by lipid lowering medication because we did not detect an association for hypercholesterolemia, where mediation use is taken into account. A 2003 study examined cardiovascular disease risk factors among end-stage renal disease patients (n = 202) [6]. Patients with IAC had higher body mass indexes and smoking history compared to patients without calcification, whereas patients with MAC did not differ from patients without calcification in these aspects [6]. Taken together, aging, male sex, statin use and history of CAD increases both IAC and MAC, while smoking is a specific risk factor for IAC and diabetes a risk factor for MAC; however, these risk factors seem to differ across different study populations.

The mechanisms for IAC and MAC are not entirely known yet. IAC is a representation of atherosclerotic plaque burden, which might be a result of vascular inflammation, while MAC may have mechanisms similar to bone formation without an inflammatory basis [23]. To date, no biomarkers have been identified that are specifically associated with IAC or MAC. Our sample for measurement of biomarkers was small and therefore our results are exploratory in nature. We hypothesized that bone markers, such as osteonectin, may serve as marker of MAC. However, we found that osteonectin was associated with a reduced risk of MAC compared to IAC, which is in line with a previous study identifying osteonectin in atherosclerotic plaques [24]. The function of osteonectin is not fully understood yet and our results may suggest that osteonectin could serve as an indicator of IAC. The medial layer of arteries mainly consists of vascular smooth muscle cells (VSMC) [25] and an in vitro study showed that VSMC treated with vitamin K antagonists resulted in increased calcification [26]. Additionally, MGP knockout mice suffered from severe medial calcification, whereas MGP overexpression in VSMC reduced medial calcification [27]. Similar associations were recently reported in women using warfarin [28]. We used the inactive dp-ucMGP as a marker of vitamin K status and found similar results compared to these previous studies, which suggest that vitamin K may specifically be involved in medial calcification. These results are also in line with a recent study of Jaminon et al. showing an association of high dp-ucMGP concentrations with high medial calcification scores in patients with chronic kidney disease [29]. However, further research is necessary to confirm these biomarkers as markers for IAC and MAC.

Our results support the hypothesis that MAC and IAC are part of different pathophysiological processes. Increasing evidence links MAC to adverse cardiovascular outcomes [2,6,7,10]. Intimal calcification may cause lumen narrowing and plaque instability and so appears to be related with acute vascular diseases [30]. Medial calcification may increase arterial stiffness, which may result in hypertension, chronic vascular disease such as peripheral arterial disease and heart failure [30]. In light of this, the distinction between MAC and IAC and their respective associated risk factors becomes relevant for future development of prevention and treatment strategies. We aim to follow these cohorts in a prospective study, to study the clinical consequences of calcification.

In conclusion, there is often a predominant arterial calcification type (IAC or MAC) throughout the lower extremity. These patterns of calcification, predominant IAC or predominant MAC, appear to have different associated risk factor profiles. Vascular consequences of these patterns need to be investigated in future studies. The distinction between IAC and MAC might be valuable in order to develop prevention and treatment strategies for both pathophysiological mechanisms.

## Supporting information

S1 Fig The points of the femoral calcification score described by predominant intimal (IAC) and medial (MAC) calcification. (DOCX)

S1 Table Calcification score. (DOCX)

S2 Table Baseline characteristics by the femoral calcification score in SMART (n = 520) and DCS cohort (n = 193). (DOCX)

S3 Table Baseline characteristics by the crural calcification score in SMART (n = 520) and DCS cohort (n = 198). (DOCX)

S4 Table Risk (OR 95%CI) of predominant intimal, predominant medial or indistinguishable calcification compared to no calcification in the crural arteries. (DOCX)

S5 Table Risk (OR 95%CI) of predominant intimal, predominant medial or indistinguishable calcification compared to no calcification in the femoral artery using a cutoff of 6 to determine medial calcification. (DOCX)

S6 Table Risk (OR 95%CI) of predominant intimal, predominant medial or indistinguishable calcification compared to no calcification in the femoral artery using cutoff 8 to determine medial calcification. (DOCX)

S7 Table Biomarkers in participants with predominant Intimal and predominant Medial calcification in the femoral artery. (DOCX)
