# Supplementary material for: Intimal and medial calcification in relation to cardiovascular risk factors
Source: PLoS One. 2020 Jul 13;15(7):e0235228. doi: 10.1371/journal.pone.0235228 (PMC7357737; doi:10.1371/journal.pone.0235228)
Supplement: S1 Fig — (DOCX) [file pone.0235228.s001.docx]

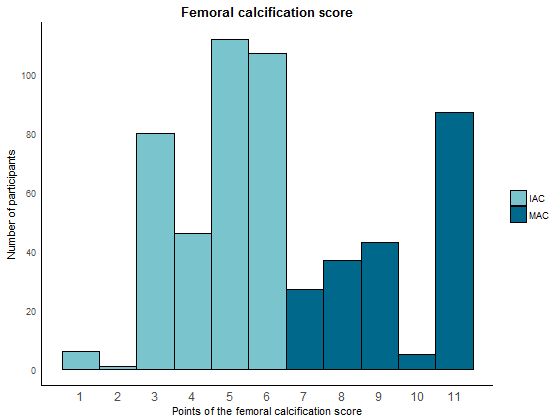


**Supplementary figure 1.** The points of the femoral calcification score described by predominant intimal (IAC) and medial (MAC) calcification.
