# Supplementary material for: Intimal and medial calcification in relation to cardiovascular risk factors
Source: PLoS One. 2020 Jul 13;15(7):e0235228. doi: 10.1371/journal.pone.0235228 (PMC7357737; doi:10.1371/journal.pone.0235228)
Supplement: S1 Table — (DOCX) [file pone.0235228.s002.docx]

**Supplementary** **table 1.** Calcification score

| **Aspect** |  | **Points** |
| --- | --- | --- |
| Circularity | Absent | 0 |
|  | Dot(s) | 1 |
|  | <90 degrees | 2 |
|  | 90-270 degrees | 3 |
|  | 270-360 degrees | 4 |
| Thickness | Absent | 0 |
|  | Thick ≥1.5 mm | 1 |
|  | Thin <1.5 mm | 3 |
| Morphology | Indistinguishable | 0 |
|  | Irregular/Patchy | 1 |
|  | Continuous | 4 |
