# Supplementary material for: Intimal and medial calcification in relation to cardiovascular risk factors
Source: PLoS One. 2020 Jul 13;15(7):e0235228. doi: 10.1371/journal.pone.0235228 (PMC7357737; doi:10.1371/journal.pone.0235228)
Supplement: S2 Table — (DOCX) [file pone.0235228.s003.docx]

| **Supplementary table 2.** Baseline characteristics by the femoral calcification score in SMART (n=520) and DCS cohort (n=193). | | | | |
| --- | --- | --- | --- | --- |
|  | *Absent (n=162)* | *Intimal*  *(n=271)* | *Medial*  *(n=199)* | *Indistinguishable*  *(n=81)* |
| Age _(years)_ | 54.1 ± 11.6 | 64.7 ± 9.2 | 65.5 ± 8.7 | 59.6 ± 8.8 |
| Male sex | 100 (62%) | 225 (83%) | 167 (84%) | 58 (72%) |
| BMI _(kg/m2)_ | 28.1 ± 4.8 | 27.8 ± 4.2 | 28.2 ± 4.6 | 28.3 ± 4.2 |
| Diabetes (type 1 and 2) | 54 (33%) | 98 (36%) | 99 (50%) | 23 (28%) |
| Hypertension | 85 (53%) | 161 (59%) | 133 (67%) | 45 (56%) |
| Hyperlipidemia | 50 (31%) | 95 (35%) | 72 (36%) | 25 (31%) |
| Systolic blood pressure _(mmHg)_ | 129 ± 15 | 134 ± 17 | 135 ± 19 | 133 ± 17 |
| Diastolic blood pressure _(mmHg)_ | 78 ± 9 | 78 ± 9 | 78 ± 9 | 80 ± 9 |
| Smoking |  |  |  |  |
| Current | 30 (19%) | 68 (26%) | 18 (9%) | 18 (22%) |
| Former | 68 (42%) | 159 (59%) | 94 (48%) | 40 (49%) |
| Never | 63 (39%) | 42 (16%) | 86 (44%) | 23 (28%) |
| High ABI _(≥1.3)_ | 36 (22%) | 31 (12%) | 55 (28%) | 21 (26%) |
| Low ABI _(≤0.9)_ | 4 (3%) | 32 (12%) | 9 (5%) | 1 (1%) |
| Statin use (yes vs no) | 107 (66%) | 240 (89%) | 165 (83%) | 65 (80%) |
| *Manifest cardiovascular disease* | |  |  |  |
| Cerebrovascular disease | 45 (28%) | 26 (10%) | 24 (12%) | 12 (15%) |
| Coronary artery disease | 62 (38%) | 180 (66%) | 120 (60%) | 46 (57%) |
| Aneurysm abdominal aorta | 3 (2%) | 16 (6%) | 6 (3%) | 1 (1%) |
| Peripheral artery disease | 8 (5%) | 20 (7%) | 5 (3%) | 4 (5%) |
|  |  |  |  |  |
| eGFR _(ml/min/1.73m2)_ | 82 ± 23 | 81 ± 19 | 77 ± 20 | 88 ± 19 |
| Triglycerides _(mmol/L)_ | 1.4 (1.0-2.2) | 1.3 (1.0-2.0) | 1.4 (1.0-1.8) | 1.3 (1.0–1.9) |
| Total cholesterol _(mmol/L)_ | 4.5 ± 1.2 | 4.3 ± 1.0 | 4.1 ± 1.0 | 4.5 ± 1.2 |
| LDL-cholesterol _(mmol/L)_ | 2.6 ± 1.0 | 2.4 ± 0.8 | 2.2 ± 0.8 | 2.5 ± 1.0 |
| HDL-cholesterol _(mmol/L)_ | 1.2 ± 0.3 | 1.2 ± 0.3 | 1.2 ± 0.3 | 1.3 ± 0.4 |
| HbA1c _(mmol/mol)_ | 42.3 ± 13.8 | 41.5 ± 10.2 | 44.9 ± 11.5 | 40.6 ± 10.2 |
| CRP _(mg/L)_ | 1.5 (0.8–3.9) | 1.8 (0.9–4.0) | 1.6 (0.8-3.4) | 1.4 (0.8-3.3) |
| Baseline characteristics are described as mean ± standard deviation, median (interquartile range) or number of participants (%).  BMI: body mass index, bp: blood pressure, ABI: ankle brachial index, eGFR: estimated glomerular filtration rate, LDL: low-density lipoprotein, HDL: high-density lipoprotein, CRP: c-reactive protein. | | | | |
