# Supplementary material for: Intimal and medial calcification in relation to cardiovascular risk factors
Source: PLoS One. 2020 Jul 13;15(7):e0235228. doi: 10.1371/journal.pone.0235228 (PMC7357737; doi:10.1371/journal.pone.0235228)
Supplement: S3 Table — (DOCX) [file pone.0235228.s004.docx]

| **Supplementary table 3.** Baseline characteristics by the crural calcification score in SMART (n=520) and DCS cohort (n=198). | | | | |
| --- | --- | --- | --- | --- |
|  | *Absent (n=215)* | *Intimal*  *(n=218)* | *Medial*  *(n=181)* | *Indistinguishable*  *(n=104)* |
| Age _(years)_ | 55.4 ± 11.2 | 64.8 ± 9.0 | 66.4 ± 8.5 | 61.8 ± 9.2 |
| Male sex | 130 (61%) | 178 (82%) | 161 (89%) | 83 (80%) |
| BMI _(kg/m2)_ | 28.4 ± 4.8 | 27.5 ± 4.0 | 28.5 ± 4.7 | 27.8 ± 3.7 |
| Diabetes (type 1 and 2) | 81 (38%) | 71 (33%) | 93 (51%) | 34 (33%) |
| Hypertension | 116 (54%) | 135 (62%) | 113 (62%) | 64 (62%) |
| Hyperlipidemia | 75 (35%) | 67 (31%) | 65 (34%) | 39 (38%) |
| Systolic blood pressure _(mmHg)_ | 130 ± 16 | 134 ± 17 | 135 ± 19 | 133 ± 15 |
| Diastolic blood pressure _(mmHg)_ | 79 ± 10 | 78 ± 9 | 77 ± 9 | 78 ± 8 |
| Smoking |  |  |  |  |
| Current | 43 (20%) | 52 (24%) | 16 (9%) | 25 (24%) |
| Former | 99 (46%) | 127 (58%) | 95 (53%) | 42 (41%) |
| Never | 71 (34%) | 39 (18%) | 69 (39%) | 36 (35%) |
| High ABI _(≥1.3)_ | 41 (19%) | 26 (12%) | 55 (31%) | 21 (20%) |
| Low ABI _(≤0.9)_ | 7 (3%) | 22 (10%) | 13 (7%) | 4 (4%) |
| Statin use | 151 (70%) | 194 (89%) | 150 (83%) | 85 (82%) |
| Manifest cardiovascular disease |  |  |  |  |
| Cerebrovascular disease | 50 (23%) | 25 (12%) | 20 (11%) | 12 (12%) |
| Coronary artery disease | 84 (39%) | 156 (72%) | 114 (63%) | 54 (52%) |
| Aneurysm abdominal aorta | 8 (4%) | 8 (4%) | 6 (3%) | 4 (4%) |
| Peripheral artery disease | 11 (5%) | 16 (7%) | 6 (3%) | 4 (4%) |
|  |  |  |  |  |
| eGFR _(ml/min/1.73m2)_ | 80 ± 23 | 86 ± 20 | 87 ± 24 | 92 ± 28 |
| Triglycerides _(mmol/L)_ | 1.4 (1.0-2.0) | 1.3 (1.0-1.8) | 1.4 (1.0-1.8) | 1.4 (1.1–2.0) |
| Total cholesterol _(mmol/L)_ | 4.5 ± 1.2 | 4.3 ± 1.0 | 4.1 ± 0.9 | 4.4 ± 1.2 |
| LDL-cholesterol _(mmol/L)_ | 2.5 ± 1.0 | 2.3 ± 0.8 | 2.2 ± 0.8 | 2.4 ± 1.0 |
| HDL-cholesterol _(mmol/L)_ | 1.2 ± 0.4 | 1.2 ± 0.3 | 1.2 ± 0.3 | 1.2 ± 0.3 |
| HbA1c _(mmol/mol)_ | 43.1 ± 13.2 | 40.7 ± 9.8 | 45.2 ± 11.9 | 41.1 ± 9.6 |
| CRP _(mg/L)_ | 1.4 (0.7–3.5) | 1.6 (0.9–3.9) | 1.8 (0.9-3.4) | 1.9 (0.9-3.9) |
| Baseline characteristics are described as mean ± standard deviation, median (interquartile range) or number of participants (%).  BMI: body mass index, bp: blood pressure, ABI: ankle brachial index, eGFR: estimated glomerular filtration rate, LDL: low-density lipoprotein, HDL: high-density lipoprotein, CRP: c-reactive protein. | | | | |
