# Supplementary material for: Intimal and medial calcification in relation to cardiovascular risk factors
Source: PLoS One. 2020 Jul 13;15(7):e0235228. doi: 10.1371/journal.pone.0235228 (PMC7357737; doi:10.1371/journal.pone.0235228)
Supplement: S4 Table — (DOCX) [file pone.0235228.s005.docx]

| **Supplementary table 4.** Risk (OR 95%CI) of predominant intimal, predominant medial or indistinguishable calcification compared to no calcification in the crural arteries. | | | | | |
| --- | --- | --- | --- | --- | --- |
|  | *Absent*  *(n=215)* | *Intimal*  *(n=218)* | *Medial*  *(n=181)* | *Indistinguishable (n=104)* | *Media vs. intima* |
| Age _(per 10 years)_ | 1 | 2.81 (2.23;3.54) | 3.55 (2.75;4.59) | 1.94 (1.51;2.51) | 1.09 (0.85;1.39) |
| Male sex | 1 | 3.79 (2.33;6.16) | 7.30 (4.04;13.20) | 3.04 (1.72;5.40) | 2.20 (1.20;4.02) |
| BMI _(per kg/m2)_ | 1 | 0.95 (0.91;1.00) | 1.02 (0.97;1.07) | 0.97 (0.92;1.02) | 1.06 (1.01;1.11) |
| Diabetes _(type 1 and 2)_ ^#^ | 1 | 0.83 (0.40;1.71) | 1.59 (0.76;3.33) | 0.46 (0.17;1.26) | 1.95 (1.08;3.52) |
| Hypertension _(yes vs no)_ | 1 | 0.96 (0.62;1.49) | 0.93 (0.58;1.49) | 1.09 (0.65;1.83) | 0.81 (0.52;1.26) |
| Hyperlipidemia _(yes vs no)_ | 1 | 0.55 (0.34;0.88) | 0.67 (0.41;1.10) | 0.93 (0.55;1.59) | 0.63 (0.33;1.22) |
| Systolic blood pressure _(per 10 mmHg)_ | 1 | 1.01 (0.89;1.15) | 1.00 (0.87;1.14) | 1.00 (0.86;1.16) | 0.97 (0.86;1.09) |
| Diastolic blood pressure _(per 10 mmHg)_ | 1 | 0.82 (0.65;1.03) | 0.70 (0.54;0.90) | 0.81 (0.62;1.05) | 0.85 (0.68;1.07) |
| Smoking _(current vs never)_ | 1 | 3.62 (1.89;6.93) | 0.62 (0.29;1.30) | 1.53 (0.77;3.06) | 0.16 (0.08;0.33) |
| Pack years ^#^ | 1 | 1.02 (1.01;1.04) | 1.00 (0.98;1.01) | 1.01 (1.00;1.03) | 0.97 (0.96;0.99) |
| High ABI _(>1.3)_ | 1 | 0.48 (0.27;0.87) | 1.50 (0.88;2.58) | 0.96 (0.52;1.79) | 3.13 (1.84;5.31) |
| Low ABI _(<0.9)_ | 1 | 3.89 (1.46;10.40) | 2.69 (0.91;7.90) | 1.41 (0.38;5.19) | 0.68 (0.33;1.43) |
| Statin use _(yes vs no)_ | 1 | 2.69 (1.52;4.76) | 1.42 (0.81;2.49) | 1.48 (0.81;2.71) | 0.53 (0.29;0.97) |
| Manifest cardiovascular disease _(yes vs no)_ | |  |  |  |  |
| Cerebrovascular disease | 1 | 0.71 (0.39;1.28) | 0.73 (0.38;1.40) | 0.64 (0.31;1.31) | 1.08 (0.57;2.05) |
| Coronary artery disease | 1 | 4.60 (2.86;7.38) | 2.91 (1.78;4.74) | 1.58 (0.95;2.65) | 0.81 (0.47;1.42) |
| Aneurysm abdominal aorta | 1 | 0.62 (0.20;1.87) | 0.49 (0.15;1.63) | 0.72 (0.20;2.61) | 0.92 (0.31;2.73) |
| Peripheral artery disease | 1 | 2.18 (0.88;5.42) | 1.06 (0.34;3.29) | 1.01 (0.30;3.42) | 0.46 (0.17;1.23) |
|  |  |  |  |  |  |
| eGFR _(ml/min/1.73m2)_ | 1 | 0.93 (0.85;1.03) | 0.95 (0.86;1.05) | 0.84 (0.75;0.94) | 1.00 (0.92;1.10) |
| Triglycerides _(mmol/L)_ | 1 | 1.00 (0.83;1.20) | 0.98 (0.80;1.19) | 0.99 (0.79;1.23) | 0.96 (0.81;1.14) |
| Total cholesterol _(mmol/L)_ | 1 | 1.02 (0.84;1.24) | 0.91 (0.73;1.14) | 1.02 (0.81;1.28) | 0.92 (0.74;1.14) |
| LDL-cholesterol _(mmol/L)_ | 1 | 0.99 (0.78;1.26) | 0.89 (0.68;1.16) | 1.02 (0.78;1.35) | 0.95 (0.73;1.25) |
| HDL-cholesterol _(mmol/L)_ | 1 | 1.59 (0.83;3.05) | 1.10 (0.53;2.29) | 1.08 (0.49;2.38) | 0.68 (0.34;1.37) |
| HbA1c _(mmol/mol)_ | 1 | 0.61 (0.48;0.77) | 0.95 (0.76;1.17) | 0.71 (0.54;0.93) | 1.45 (1.13;1.87) |
| CRP _(mg/L)_ | 1 | 1.00 (0.96;1.05) | 1.01 (0.97;1.06) | 1.01 (0.96;1.05) | 1.01 (0.98;1.04) |
| Every line of this table represents a separate multinomial model. All models are adjusted for age and sex.  BMI: body mass index, bp: blood pressure, ABI: ankle brachial index, eGFR: estimated glomerular filtration rate,  LDL: low-density lipoprotein, HDL: high-density lipoprotein, CRP: c-reactive protein.  ^#^ assessed in the SMART cohort only (intimal n=208, medial n=133, indistinguishable n=66). | | | | | |
