# Supplementary material for: Intimal and medial calcification in relation to cardiovascular risk factors
Source: PLoS One. 2020 Jul 13;15(7):e0235228. doi: 10.1371/journal.pone.0235228 (PMC7357737; doi:10.1371/journal.pone.0235228)
Supplement: S5 Table — (DOCX) [file pone.0235228.s006.docx]

| **Supplementary Table 5.** Risk (OR 95%CI) of predominant intimal, predominant medial or indistinguishable calcification compared to no calcification in the femoral artery using a cutoff of 6 to determine medial calcification. | | | | | |  |
| --- | --- | --- | --- | --- | --- | --- |
|  | *Absent*  *(n=162)* | *Intimal*  *(n=164)* | *Medial*  *(n=306)* | *Indistinguishable (n=81)* | *Media vs. Intimal* | |
| Age _(per 10 years)_ | 1 | 2.75 (2.14;3.54) | 3.44 (2.71;4.37) | 1.68 (1.28;2.20) | 1.15 (0.92;1.45) | |
| Male sex | 1 | 3.47 (2.00;6.03) | 4.10 (2.49;6.77) | 1.70 (0.94;3.06) | 1.26 (0.75;2.12) | |
| BMI _(perkg/m2)_ | 1 | 0.95 (0.90;1.01) | 1.02 (0.97;1.07) | 1.01 (0.95;1.07) | 1.06 (1.01;1.11) | |
| Diabetes _(type 1 and 2)_ ^#^ | 1 | 1.12 (0.49;2.53) | 2.02 (0.88;4.64) | 0.93 (0.33;2.63) | 1.81 (1.03;3.15) | |
| Hypertension _(yes vs no)_ | 1 | 0.83 (0.51;1.36) | 1.10 (0.70;1.74) | 0.90 (0.51;1.59) | 1.21 (0.80;1.83) | |
| Hyperlipidemia _(yes vs no)_ | 1 | 0.85 (0.50;1.45) | 0.92 (0.57;1.50) | 0.86 (0.46;1.59) | 0.72 (0.41;1.25) | |
| Systolic blood pressure _(per 10 mmHg)_ | 1 | 1.00 (0.86;1.16) | 1.03 (0.90;1.18) | 1.08 (0.91;1.28) | 1.02 (0.90;1.14) | |
| Diastolic blood pressure _(per 10 mmHg)_ | 1 | 0.91 (0.70;1.17) | 0.89 (0.70;1.12) | 1.17 (0.88;1.55) | 0.97 (0.78;1.20) | |
| Smoking _(current vs never)_ | 1 | 7.57 (3.59;15.99) | 1.40 (0.72;2.72) | 2.12 (0.96;4.71) | 0.17 (0.09;0.33) | |
| Pack years ^#^ | 1 | 1.04 (1.02;1.06) | 1.00 (0.98;1.02) | 1.03 (1.00;1.05) | 0.96 (0.95;0.98) | |
| High ABI _(>1.3)_ | 1 | 0.26 (0.13;0.53) | 0.76 (0.45;1.30) | 1.07 (0.57;2.04) | 2.84 (1.56;5.17) | |
| Low ABI _(<0.9)_ | 1 | 5.91 (1.72;20.32) | 4.31 (1.27;14.65) | 0.58 (0.06;5.45) | 0.75 (0.39;1.46) | |
| Statin use _(yes vs no)_ | 1 | 3.73 (1.94;7.16) | 2.10 (1.24;3.53) | 1.81 (0.94;3.49) | 0.60 (0.33;1.11) | |
| Manifest cardiovascular disease _(yes vs no)_ | |  |  |  |  | |
| Cerebrovascular disease | 1 | 0.49 (0.26;0.94) | 0.49 (0.27;0.87) | 0.57 (0.28;1.18) | 1.37 (0.76;2.48) | |
| Coronary artery disease | 1 | 3.43 (2.05;5.75) | 2.72 (1.71;4.34) | 2.06 (1.16;3.67) | 1.03 (0.62;1.72) | |
| Aneurysm abdominal aorta | 1 | 2.55 (0.56;11.59) | 2.32 (0.54;10.08) | 0.68 (0.07;6.94) | 0.99 (0.40;2.44) | |
| Peripheral artery disease | 1 | 2.33 (0.82;6.62) | 1.38 (0.48;3.93) | 1.28 (0.36;4.59) | 0.61 (0.27;1.38) | |
|  |  |  |  |  |  | |
| eGFR _(ml/min/1.73m2)_ | 1 | 1.02 (0.92;1.14) | 0.93 (0.85;1.03) | 0.97 (0.86;1.10) | 0.89 (0.82;0.98) | |
| Triglycerides _(mmol/L)_ | 1 | 0.93 (0.76;1.12) | 0.87 (0.73;1.05) | 0.77 (0.58;1.03) | 0.93 (0.79;1.10) | |
| Total cholesterol _(mmol/L)_ | 1 | 1.06 (0.85;1.32) | 0.88 (0.72;1.08) | 1.01 (0.79;1.29) | 0.84 (0.69;1.02) | |
| LDL-cholesterol _(mmol/L)_ | 1 | 1.06 (0.81;1.37) | 0.84 (0.65;1.07) | 1.03 (0.77;1.38) | 0.81 (0.63;1.03) | |
| HDL-cholesterol _(mmol/L)_ | 1 | 2.38 (1.09;5.81) | 2.03 (0.98;4.21) | 2.75 (1.17;6.44) | 0.91 (0.49;1.69) | |
| HbA1c _(mmol/mol)_ | 1 | 0.97 (0.95;0.99) | 0.99 (0.97;1.01) | 0.74 (0.55;1.00) | 1.01 (0.99;1.03) | |
| CRP _(mg/L)_ | 1 | 1.01 (0.97;1.04) | 0.99 (0.95;1.03) | 0.96 (0.90;1.03) | 0.99 (0.96;1.02) | |
| Every line of this table represents a separate multinomial model. All models are adjusted for age and sex.  BMI: body mass index, bp: blood pressure, ABI: ankle brachial index, eGFR: estimated glomerular filtration rate,  LDL: low-density lipoprotein, HDL: high-density lipoprotein, CRP: c-reactive protein, AAA: Aneurysm abdominal aorta.  ^#^ assessed in the SMART cohort only | | | | | |  |
