# Supplementary material for: Intimal and medial calcification in relation to cardiovascular risk factors
Source: PLoS One. 2020 Jul 13;15(7):e0235228. doi: 10.1371/journal.pone.0235228 (PMC7357737; doi:10.1371/journal.pone.0235228)
Supplement: S6 Table — (DOCX) [file pone.0235228.s007.docx]

| **Supplementary Table 6.** Risk (OR 95%CI) of predominant intimal, predominant medial or indistinguishable calcification compared to no calcification in the femoral artery using cutoff 8 to determine medial calcification. | | | | | |  |
| --- | --- | --- | --- | --- | --- | --- |
|  | *Absent*  *(n=162)* | *Intimal*  *(n=298)* | *Medial*  *(n=172)* | *Indistinguishable (n=81)* | *Media vs. Intimal* | |
| Age _(per 10 years)_ | 1 | 2.99 (2.37;3.77) | 3.51 (2.28;7.11) | 1.68 (1.28;2.20) | 1.07 (0.85;1.34) | |
| Male sex | 1 | 3.75 (2.30;6.13) | 4.03 (2.28;7.11) | 1.70 (0.94;3.06) | 1.16 (0.69;1.96) | |
| BMI _(perkg/m2)_ | 1 | 0.99 (0.94;1.04) | 1.01 (0.96;1.07) | 1.01 (0.95;1.07) | 1.01 (0.97;1.06) | |
| Diabetes _(type 1 and 2)_ ^#^ | 1 | 1.18 (0.53;2.66) | 2.05 (0.87;4.79) | 0.46 (0.17;1.26) | 1.72 (0.98;3.03) | |
| Hypertension _(yes vs no)_ | 1 | 0.92 (0.59;1.44) | 1.13 (0.69;1.88) | 0.90 (0.51;1.59) | 1.10 (0.72;1.67) | |
| Hyperlipidemia _(yes vs no)_ | 1 | 0.86 (0.53;1.40) | 0.96 (0.56;1.62) | 0.86 (0.46;1.59) | 0.66 (0.37;1.19) | |
| Systolic blood pressure _(per 10 mmHg)_ | 1 | 1.02 (0.90;1.17) | 1.02 (0.88;1.18) | 1.08 (0.91;1.28) | 0.98 (0.87;1.10) | |
| Diastolic blood pressure _(per 10 mmHg)_ | 1 | 0.95 (0.75;1.19) | 0.79 (0.61;1.04) | 1.17 (0.88;1.55) | 0.83 (0.67;1.04) | |
| Smoking _(current vs never)_ | 1 | 6.47 (3.28;12.79) | 0.74 (0.35;1.58) | 2.12 (0.96;4.71) | 0.11 (0.05;0.21) | |
| Pack years ^#^ | 1 | 1.04 (1.02;1.06) | 1.00 (0.97;1.02) | 1.03 (1.00;1.05) | 0.96 (0.94;0.97) | |
| High ABI _(>1.3)_ | 1 | 0.33 (0.18;0.58) | 1.11 (0.63;1.95) | 1.07 (0.57;2.04) | 3.31 (2.03;5.41) | |
| Low ABI _(<0.9)_ | 1 | 6.08 (1.84;20.11) | 2.78 (0.73;10.55) | 0.58 (0.06;5.45) | 0.47 (0.22;1.02) | |
| Statin use _(yes vs no)_ | 1 | 3.10 (1.81;5.34) | 1.85 (1.03;3.29) | 1.81 (0.94;3.49) | 0.64 (0.37;1.10) | |
| Manifest cardiovascular disease _(yes vs no)_ | |  |  |  |  | |
| Cerebrovascular disease | 1 | 0.43 (0.24;0.79) | 0.57 (0.31;1.07) | 0.57 (0.28;1.18) | 1.37 (0.76;2.48) | |
| Coronary artery disease | 1 | 3.31 (2.06;5.31) | 2.54 (1.54;4.17) | 2.06 (1.16;3.67) | 0.93 (0.57;1.52) | |
| Aneurysm abdominal aorta | 1 | 3.05 (0.72;12.90) | 1.49 (0.30;7.27) | 0.68 (0.07;6.94) | 0.52 (0.20;1.37) | |
| Peripheral artery disease | 1 | 2.42 (0.90;6.50) | 0.80 (0.23;2.80) | 1.28 (0.36;4.59) | 0.34 (0.12;0.92) | |
|  |  |  |  |  |  | |
| eGFR _(ml/min/1.73m2)_ | 1 | 0.98 (0.88;1.08) | 0.95 (0.85;1.06) | 0.97 (0.86;1.10) | 0.96 (0.89;1.04) | |
| Triglycerides _(mmol/L)_ | 1 | 0.91 (0.77;1.08) | 0.86 (0.70;1.06) | 0.77 (0.58;1.03) | 0.93 (0.77;1.12) | |
| Total cholesterol _(mmol/L)_ | 1 | 0.99 (0.81;1.21) | 0.86 (0.69;1.09) | 1.01 (0.79;1.29) | 0.89 (0.73;1.08) | |
| LDL-cholesterol _(mmol/L)_ | 1 | 0.97 (0.76;1.23) | 0.83 (0.63;1.10) | 1.03 (0.77;1.38) | 0.89 (0.70;1.14) | |
| HDL-cholesterol _(mmol/L)_ | 1 | 2.19 (1.07;4.49) | 2.11 (0.95;4.67) | 2.75 (1.17;6.44) | 1.02 (0.55;1.89) | |
| HbA1c _(mmol/mol)_ | 1 | 0.97 (0.95;0.99) | 1.00 (0.98;1.02) | 0.74 (0.55;1.00) | 1.03 (1.01;1.06) | |
| CRP _(mg/L)_ | 1 | 1.00 (0.96;1.04) | 0.99 (0.95;1.03) | 0.96 (0.90;1.03) | 0.99 (0.96;1.02) | |
| Every line of this table represents a separate multinomial model. All models are adjusted for age and sex.  BMI: body mass index, bp: blood pressure, ABI: ankle brachial index, eGFR: estimated glomerular filtration rate,  LDL: low-density lipoprotein, HDL: high-density lipoprotein, CRP: c-reactive protein, AAA: Aneurysm abdominal aorta.  ^#^ assessed in the SMART cohort only | | | | | |  |
