# Supplementary material for: Intimal and medial calcification in relation to cardiovascular risk factors
Source: PLoS One. 2020 Jul 13;15(7):e0235228. doi: 10.1371/journal.pone.0235228 (PMC7357737; doi:10.1371/journal.pone.0235228)
Supplement: S7 Table — (DOCX) [file pone.0235228.s008.docx]

| **Supplementary table 7.** Biomarkers in participants with predominant Intimal and predominant Medial calcification in the femoral artery. | | | | |
| --- | --- | --- | --- | --- |
|  | Femoral calcification | | Crural calcification | |
|  | **Dominant Intimal (n=151)** | **Dominant Medial (n=151)** | **Dominant Intimal (n=151)** | **Dominant Medial (n=120)** |
| *Vitamin K markers* |  |  |  |  |
| Dp-ucMGP _(pmol/L)_ | 466 (469-742) | 685.9 (495-853) | 567 (469-739) | 646.5 (517-882) |
| PIVKA II _>0.25 AU/ml*_ | 19 (6.3%) | 22 (7.3%) | 19 (7.0%) | 18 (6.6%) |
| *Bone markers* |  |  |  |  |
| Osteocalcin _(ng/ml)_ | 30.5 (23-44.3) | 29 (21-40.3) | 31 (23-44) | 29 (22-40) |
| Osteonectin _(ng/ml)_ | 1028 (887-1145) | 1005 (838-1125) | 1026 (886-1143) | 1009 (838-1124) |
| Osteopontin _(ng/ml)_ | 17 (13-22) | 16 (13-22) | 17 (13-22) | 16 (13-22) |
| *Inflammatory markers* |  |  |  |  |
| IL-6 _(pg/ml)_ | 0.7 (0.47-1.07) | 0.6 (0.43-0.87) | 0.71 (0.47-1.08) | 0.64 (0.47-0.89) |
| IL-8 _(pg/ml)_ | 12.4 (8.6-17.47 | 11.95 (8.28-20.1) | 12.5 (8.6-17.7) | 11.9 (8.2-18.5) |
| TNF-alfa _(pg/ml)_ | 2.05 (1.7-2.5) | 2.2 (1.7-2.8) | 2.1 (1.7-2.5) | 2.3 (1.7-2.8) |
| Median and interquartile ranges or n (percentage).  Dp-ucMGP: desphosphorylated-uncarboxylated Matrix Gla Protein, PIVKA-II: protein induced by vitamin K absence/antagonism II, IL: interleukin  * PIVKA is measured as detectable/non detectable with a detection limit of 0.25 AU/ml | | | | |
